# Distribution of short interstitial telomere motifs in two plant genomes: putative origin and function

**DOI:** 10.1186/1471-2229-10-283

**Published:** 2010-12-20

**Authors:** Christine Gaspin, Jean-François Rami, Bernard Lescure

**Affiliations:** 1 INRA Toulouse, UBIA & Plateforme Bioinformatique, UR 875, Chemin de Borde Rouge, Auzeville BP 52627, 31326 Castanet-Tolosan, France; 2 Centre de coopération internationale en recherche agronomique pour le développement (CIRAD). UMR Développement et Amélioration des Plantes, TA A96/3, Avenue Agropolis, 34398 Montpellier Cedex 5, France; 3 Laboratoire Interactions Plantes-Microorganismes (LIPM), UMR 441-2594 (INRA-CNRS), BP 52627, Chemin de Borde Rouge, Auzeville BP 52627, 31326 Castanet-Tolosan, France

## Abstract

**Background:**

Short interstitial telomere motifs (*telo *boxes) are short sequences identical to plant telomere repeat units. They are observed within the 5' region of several genes over-expressed in cycling cells. In synergy with various *cis*-acting elements, these motifs participate in the activation of expression. Here, we have analysed the distribution of *telo *boxes within *Arabidopsis thaliana *and *Oryza sativa *genomes and their association with genes involved in the biogenesis of the translational apparatus.

**Results:**

Our analysis showed that the distribution of the *telo *box (AAACCCTA) in different genomic regions of *A. thaliana *and *O. sativa *is not random. As is also the case for plant microsatellites, they are preferentially located in the 5' flanking regions of genes, mainly within the 5' UTR, and distributed as a gradient along the direction of transcription. As previously reported in *Arabidopsis*, a conserved topological association of *telo *boxes with site II or TEF *cis*-acting elements is observed in almost all promoters of genes encoding ribosomal proteins in *O. sativa*. Such a conserved promoter organization can be found in other genes involved in the biogenesis of the translational machinery including rRNA processing proteins and snoRNAs. Strikingly, the association of *telo *boxes with site II motifs or TEF boxes is conserved in promoters of genes harbouring snoRNA clusters nested within an intron as well as in the 5' flanking regions of non-intronic snoRNA genes. Thus, the search for associations between *telo *boxes and site II motifs or TEF box in plant genomes could provide a useful tool for characterizing new cryptic RNA pol II promoters.

**Conclusions:**

The data reported in this work support the model previously proposed for the spreading of *telo *boxes within plant genomes and provide new insights into a putative process for the acquisition of microsatellites in plants. The association of *telo *boxes with site II or TEF *cis*-acting elements appears to be an essential feature of plant genes involved in the biogenesis of ribosomes and clearly indicates that most plant snoRNAs are RNA pol II products.

## Background

Regulatory sequences constitute a small fraction of eukaryotic genomes that determine the level, location and chronology of gene expression. In parallel to functional studies, computational analysis provides different approaches for scanning genomic sequence to identify those regions predicted to participate in gene regulation [1,2]: (i) sequence analysis of co-regulated genes within a given species, (ii) inter-species sequence comparison of orthologous genes and (iii), database construction and analysis of known transcription-factor binding sites.

Functional studies conducted to identify *trans *and *cis*-acting elements controlling the expression of translation factors and ribosomal proteins (*rp*) in *Arabidopsis *allowed us to characterize several *cis*-acting elements. One of them, the *telo *box (AAACCCTA), was first observed within the promoter of the four *Arabidopsis *genes encoding the translation elongation factor *EF1*αpromoters [3,4] and subsequently within a few plant *rp *promoters [5]. This short motif is identical to the repeat (AAACCCT)n of plant telomeres [6] but differs from long interstitial telomere repeats (ITRs) which are found at discrete intrachromosomal sites in many eukaryotic species [7,8] and probably result from chromosomal rearrangements such as end-fusions and segmental duplications. In contrast to the limited number of ITRs observed in pericentromeric and subtelomeric regions in *Arabidopsis *[8], a preliminary computational analysis suggested that short telomere repeats (*telo *boxes) were over-represented at the 5' end of *Arabidopsis *ESTs [9]. More recently, with the achievement of the *Arabidopsis *sequencing project, we showed that the occurrence of *telo *boxes within *rp *promoters is the rule rather than the exception [10,11]. *Telo *boxes were also observed in promoters of several protein-encoding genes which, as is the case for *rp*, are expected to be over-expressed in cycling cells, suggesting that it could be involved in the coordinated expression of this class of genes. Experimental data indicated that the *telo *box was indeed involved in the expression in cycling cells [11-13]. However, by itself this motif is not able to activate the transcription by RNA pol II but acts in synergy with various *cis*-acting elements to increase the expression. These *cis*-acting elements include the TEF1 box identified in promoters of the translation elongation factor EF1α[14], the Trap1 box in the promoter of a *rp *gene [15] and redundant site II motifs initially characterized in the promoter of the proliferating cellular nuclear antigen gene (PCNA) [16] and subsequently in most *Arabidopsis rp *genes [11].

In this study, we analysed the distribution of *telo *boxes within *A. thaliana *and *O. sativa *genomes and their association with genes involved in the biogenesis of the translational apparatus. In addition, this analysis revealed a striking analogy with the genomic distribution of *telo *boxes and plant microsatellites.

## Results

### Definition of the telo box and distribution in different genomic regions

**Figure 1:**
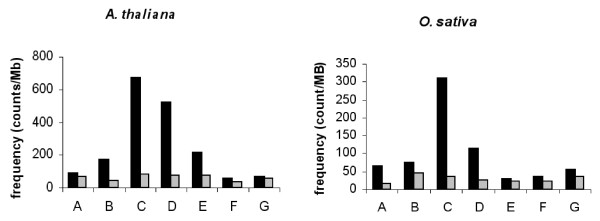
**Analysis from a circular permutation of frequencies of plant telomere motifs within 5' UTR regions**. The telomere motifs (one telomere repeat unit + one nucleotide) found in *A. thaliana *and *O. sativa *are shown in black, a control sequence in grey. **A**, CTAAACCC and TCAAACCT; **B**, TAAACCCT and CAAACCTC; **C**, AAACCCTA and AAACCTCA; **D**, AACCCTAA and AACCTCAA; **E**, ACCCTAAA and ACCTCAAA; **F**, CCCTAAAC and CCTCAAAC; **G**, CCTAAACC and CTCAAACC.

**Figure 2:**
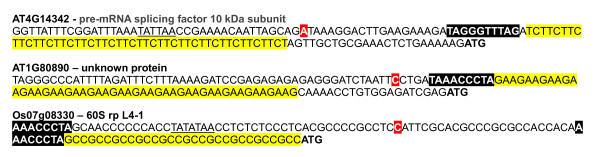
**Examples of the presence of telo boxes and trinucleotide repeats in 5' UTR of rp genes**. Occurrence of both *telo *boxes (AAACCCTA) and tri-nucleotide repeats (GAA/TTC in *Arabidopsis *and GCC/GGC in *O. sativa*) within 5'UTR. The *telo *boxes are boxed in black, the tri-nucleotide repeats in yellow, the transcription start site in red; the translation ATG codons are in bold and the putative TATA boxes are underlined.

An initial statistical study [9] conducted by using a large set of *Arabidopsis *ESTs [17,18] and *Arabidopsis *genes available at this time suggested that the sequence AAACCCTAA corresponding to 1.3 units of the plant telomere repeat AAACCCT [6] was over-represented and preferentially located in the 5' region of genes. The completion of *Arabidopsis *and *O. sativa *sequencing means that they can now be subjected to similar but exhaustive analysis. A chi-square test was used to determine whether the observed frequencies (counts) of telobox in the different compartments markedly differ from the frequencies that we would expect by chance. Chi-square statistics for *A. thaliana *and *O. sativa *were obtained that clearly indicate that the observed frequencies in each compartment differ markedly from the expected frequencies (Table 1). We also studied the occurrence of seven putative telomere motifs obtained from a circular permutation of the sequence AAACCCTA corresponding to 1.14 telomere repeat units [6]. This study was conducted by using *Arabidopsis *and *O. sativa *5' UTR sequences. The results reported in Figure 1 and Table 1 confirm our previous observations and extend them to a monocot. Among the seven sequences analysed, the motif AAACCCTA (*telo *box) is over-represented in both *Arabidopsis *and rice. The use of a control-related sequence (AAACCTCA) enabled us to exclude the base composition as a cause of the over-representation of *telo *boxes. We characterized the occurrence of *telo *boxes among the different genomic regions in the *Arabidopsis *and *O. Sativa *genomes. Just as a high level of *telo *boxes was initially observed at the 5' end of *Arabidopsis *ESTs [9], it was obvious that the frequency of *telo *boxes was higher within the 5' flanking regions, mainly within the 5' UTRs (Figure 2).

**Table 1:** Distribution of telo boxes in A. thaliana and O. sativa genomes

Genome compartment	Size	Telo counts	Telo Freq. (nb/Mb)	Telo expected	**χ**^**2**^	P	**χ**^**2**^	P
*A. thaliana*								

Genome	135709386	21057	155.2					
5'UTR	3614786	2426	680.3	561	6372	**0.E+00**	**8381**	**0,00E+000**
3'UTR	6019104	527	87	934	186	**3.E-42**		
Intron	25425536	3829	150.7	3945	4	**4.E-02**		
CDS	39588516	2966	74.9	6143	2319	**0.E+00**		
Other	61061444	11309	185.2	9474	646	**2.E-142**		

*O. sativa*								

Genome	378522865	30686	81.1					
5'UTR	7907129	2463	311.5	641	5289	**0.E+00**	**13143**	**0,00E+000**
3'UTR	15330979	460	30	1243	514	**9.E-114**		
Intron	102300755	7367	72	8293	142	**1.E-32**		
CDS	91775879	1489	16/02/10	7440	6284	**0.E+00**		
Other	161208123	18907	117.3	13069	4543	**0.E+00**		

Number of telo box motifs in the different compartments (5'UTR, 3'UTR, Introns, CDS) of *A. thaliana *and *O. sativa *genomes. A chi-square test was performed to assess deviation from the expected uniform distribution.

### Comparative distribution of telo boxes and microsatellites

**Figure 3:**
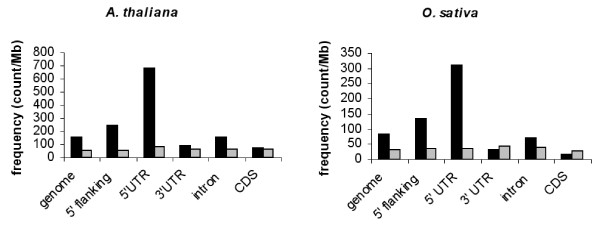
**Distribution of *telo *boxes in different genomic regions in *Arabidopsis *and *O*. *sativa***. The *telo *box, AAACCCTA, and the related sequence, AAACCTCA, are shown in black and grey, respectively.

**Figure 4:**
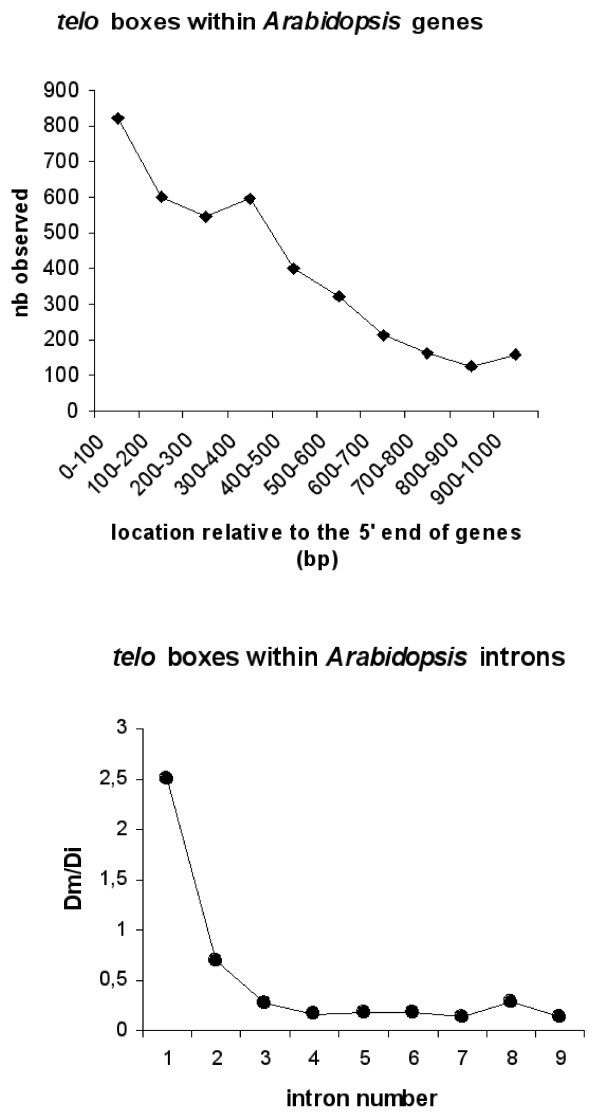
**Distribution gradient of *telo *boxes along the direction of transcription in *Arabidopsis***. Location of *telo *boxes within *Arabidopsis *genes is estimated from the TAIR database (TAIR9 CDS+UTRs+introns datasets); frequency of appearance of *telo *boxes within *Arabidopsis *introns from the TAIR9 introns datasets. Dm is the % of motifs found within a given intron relative to the total number of motifs observed within the *Arabidopsis *introns (TAIR database, introns). Di is the % of introns at a given position (intron 1, 2, 3...) relative to the estimated total number of introns.

Previous studies have revealed that in *Arabidopsis *as in *O. sativa*, microsatellites or simple sequence repeats (SSRs) and pyrimidine patches (Y Patches) are more frequently observed in 5' UTRs than in coding regions or 3' UTRs [19-24]. Among SSRs, tri-nucleotide repeats (TNRs) are more abundant and differentially represented in monocots and dicots. Thus, the TNR (GCC/GGC)n is the most abundant in the 5' flanking regions in *O. sativa *whereas it is (GAA/TTC)n in *Arabidopsis*. In contrast, Y Patches which are more frequently found in plant core promoter regions are observed in both *Arabidopsis *and *O. sativa *5' regions [22,23]. The results reported in Table 1 and Table 2 reveal a striking analogy in the genomic distribution of *telo *boxes, TNRs and Y Patches between 5' UTRs and 3' UTRs in *Arabidopsis *and *O. sativa*. The frequency of appearance of *telo *boxes is 10-20 higher within 5'UTR compared to that observed within 3'UTR. Two relevant examples of such a location of telo boxes and trinucleotide repeats in the 5' flanking regions of *Arabidopsis *and *O. sativa *rp genes are shown in Figure 2. Moreover, as has been reported for *Arabidopsis *microsatellites [19], there is a distribution gradient of *telo *boxes along the direction of transcription. The *telo *boxes (which are observed at a lower frequency within *Arabidopsis *CDS and introns - see Figure 3) are not uniformly distributed. There is a progressive decrease in the number of *telo *box motifs observed within the first 1000 nucleotides from the 5' end of genes and a higher occurrence of this motif within the first two introns (Figure 4).

**Table 2:** Distribution of telo boxes, microsatellites and Y Patch in 5' and 3' UTR in A. thaliana and O. sativa

Motif	5' UTR(number)	3' UTR (number)	5' UTR frequency counts/Mb	3' UTR frequency counts/Mb
*A. thaliana*				

AAACCCTA	2426	527	680	87
AAACCTCA	343	397	95	66
(GAA/TTC)_6_	394	49	109	8
(GCC/GGC)_6_	1	0	0.3	0
(Y/R)_18_	5216	1448	1934	322

*O. sativa*				

AAACCCTA	2463	460	311	30
AAACCTCA	278	642	35	41
(GAA/TTC)_6_	72	36	9	2
(GCC/GGC)_6_	546	25	69	2
(Y/R)_18_	6729	1827	851	119

Bytes searched: Arabidopsis 5' UTR, 3614786 bp; Arabidopsis 3' UTR, 6019104 bp; O. sativa 5' UTR, 7907129 bp; O. sativa 3' UTR, 15330979 bp.

### Telo boxes in the promoters of plant genes involved in ribosome biogenesis

**Figure 5:**
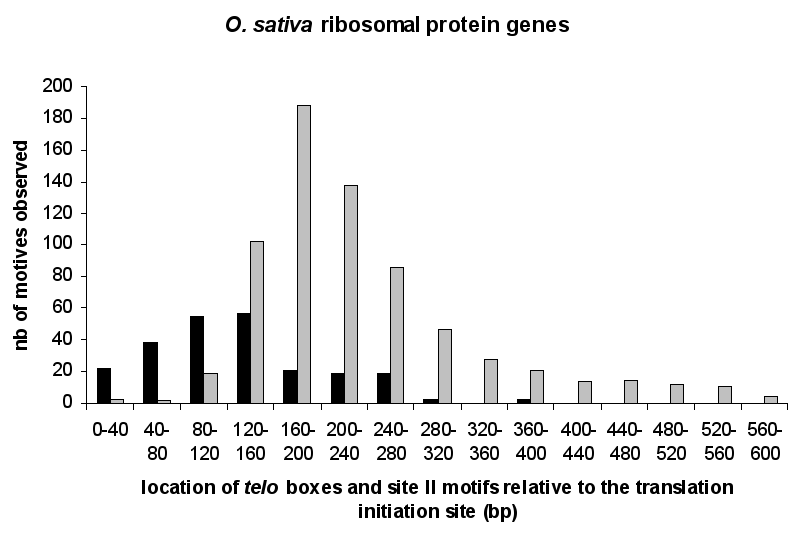
**Statistical distribution of motifs in the 5' flanking regions of *O*. *sativa *ribosomal protein genes**. Statistical distribution of *telo *boxes (black) and site II motifs (grey) in the 5' flanking regions of *O. sativa *ribosomal protein genes.

**Figure 6:**
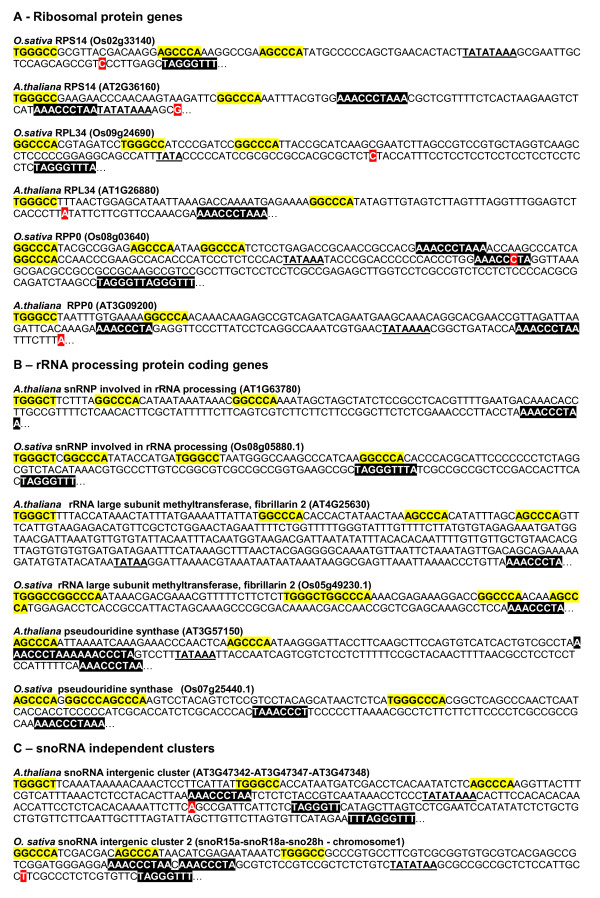
**Topological association of *telo *boxes and site II motif in 5' regions of known genes**. Illustration of the conserved topological association of *telo *boxes and site II motifs in the promoter of *Arabidopsis *and *O. sativa *orthologous ribosomal and rRNA processing protein coding genes and in the 5' flanking regions of *Arabidopsis *and *O. sativa *independently transcribed snoRNA clusters. Site II motifs are boxed in yellow, *telo *boxes in black, the location of TSS in red; putative TATA boxes are underlined.

As estimated by using the 'TAIR9 Loci Upstream Sequences -500 bp (DNA)' and 'TAIR9 5' UTRs (DNA)' datasets, the number of *Arabidopsis *genes harbouring one or several *telo *boxes within their 5' flanking region or 5' UTRs is 3234 (9.7% of *Arabidopsis *genes) and 2247 (9.2%), respectively. Among them, we have reported that ribosomal protein (*rp*) genes constituted an important sub-family showing a specific topological association of *telo *boxes with redundant site II motifs (TGGGCY) or to a lesser extent with TEF1 box (ARGGRYNNNNNGYA) *cis*-acting elements [11]. An analysis for functional categorization by loci of *Arabidopsis *genes showing an association of a *telo *box with at least two site II motifs confirms this previous observation: the product of 17.9% of these genes was expected to be associated with ribosomes against 2% for all GO annotated *Arabidopsis *genes. Here we extended this study to the monocot *O. sativa *by using the 'Ribosomal Protein Gene Database' (RPG) [24]. Out of 252 rice ribosomal protein genes, 209 (83%) contain at least one *telo *box within their 5' flanking region and 202 (80%) an association of *telo *boxes with site II motifs or TEF boxes (Additional File 1). Figure 5 shows the topological distribution of these elements. This distribution is similar to that observed for *rp *genes in Arabidopsis [11]. An illustration of this conserved lay-out within the promoter of *Arabidopsis *and rice *rp *orthologous genes is given in Figure 6A, where *telo *boxes and site II motifs are found within windows between '0 and 280 bp' and '80 and 400 bp' relative to the translation initiation codon, respectively.

In addition to ribosomal proteins, the biogenesis of cytoplasmic ribosomes also requires the biosynthesis of 5.8 S, 18 S and 25/26 S rRNAs, a process which is achieved by the transcription of rDNA and by endo- and exonucleolytic cleavages and extensive modifications of an rRNA precursor (pre-rRNA). Small nucleolar RNAs (snoRNAs), in association with specific nucleolar proteins (SnRNP), are involved in this process.

The occurrence of *telo *boxes and their association with site II motifs or TEF boxes in the promoter of genes encoding rRNA processing proteins was examined in *Arabidopsis*. For 49 genes annotated in the TAIR database as encoding a cytoplasmic rRNA processing protein, 46 (92%) contain at least one *telo *box in the 5' flanking region and 35 (70%) an association between *telo *boxes and site II motifs or TEF1 boxes (Additional File 2A and illustrations in Figure 6B). The occurrence of *telo *boxes in the 5' flanking region of *O. sativa *orthologous genes of the 46 *Arabidopsis *genes harbouring a *telo *box was analysed. By using the greenphyl database [25] we identified 37 orthologous rice genes. For 30 of them (81%), at least one *telo *box was identified within the 1 Kb 5' flanking region and for 25 (68%) an association of *telo *boxes with site II motifs or a TEF box was observed (Additional File 2B and illustrations in Figure 6B). The same analysis was conducted for snoRNA genes in *Arabidopsis *and *O. sativa*. The resulting data are summarized in Table 3. In *Arabidopsis *there are 71 snoRNA genes annotated in the TAIR database. These snoRNA genes are orphans or associated in clusters. Three of them are nested within introns of genes containing a typical association of *telo *boxes and site II motifs within their promoters (Additional File 3). For the remaining 40 non-intronic loci, a search for the occurrence of *telo *boxes, site II motifs and TEF1 boxes was carried out upstream from the 5' end of the far-upstream mature snoRNA. For 37 loci (92%) *telo *boxes were observed and for 34 (85%) an association of *telo *boxes with site II motifs or TEF1 boxes (Additional File 3 and illustration in Figure 5C). In *O. sativa *the analysis was conducted on 109 putative snoRNA loci comprising 67 clusters and 42 orphan snoRNA genes. The detail of this analysis is shown in Additional File 4. As previously reported [26,27], intronic snoRNA loci are more frequent in rice than in *Arabidopsis*. In the present work they were estimated at 31 (28% of snoRNA loci). 15 of the clusters or orphan intronic snoRNA genes are nested within introns of *rp *genes showing an association of *telo *boxes with site II motifs within their promoter. For 10 of the 16 remaining intronic snoRNA genes a similar association was observed. The analysis of 5' flanking sequences of independent snoRNA clusters confirms the data obtained for *Arabidopsis*: out of 41 independent clusters, 22 (54%) harbour a *telo *box within the 5' flanking region and 21 (51%) an association of *telo *boxes with site II motifs (Additional File 5). This conservation is less evident for non-intronic orphan snoRNA genes but remains significant: out of 35 non-intronic orphan genes, 15 (43%) contain a *telo *box and 14 (40%) an association of *telo *boxes with site II motifs within the 5' flanking sequences. To summarize, 57% of *O. sativa *snoRNA putative loci studied in this work contain at least one *telo *box and 56% an association of *telo *boxes with site II motifs in their 5' flanking region. As discussed, the loci which are not associated with *telo *boxes and site II motifs could be transcribed by RNA pol III or pseudogenes.

**Table 3:** Summary of the analysis of 5' flanking regions of A. thaliana and O. sativa snoRNA genes

	Analysed (Number)	*telo *boxes	Associations *telo *box - sites II	Associations *telo *box - TEF
*A. thaliana*				

Intronic snoRNA clusters	1	1	1	-
Intronic orphan snoRNAs	2	2	1	1
Intergenic snoRNA clusters	17	16	16	1
Intergenic orphan snoRNAs	23	21	17	1

*O. sativa*				

Intronic snoRNA clusters	25	22	22	1
Intronic orphan snoRNAs	7	5	3	0
Intergenic snoRNA clusters	42	20	19	1
Intergenic orphan snoring	47	13	8	0

For details see text and data reported in Additional Files 3 and 4.

### Identification of cryptic promoters by using the conserved topological association of telo boxes with cis-acting elements

**Figure 7:**
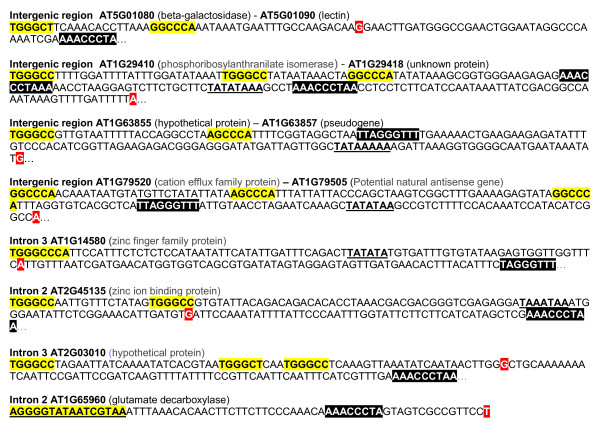
**Use of the conserved topological association of motifs to characterize cryptic RNA pol II promoters**. Site II motifs are boxed in yellow, TEF1 boxes in yellow and underlined, *telo *boxes in black, TSS in red; putative TATA boxes are underlined.

As illustrated by the characterization of unknown snoRNA gene promoters, the use of the conserved topological association of telo boxes with cis-acting elements observed within promoters of genes involved in ribosome biogenesis could provide an interesting tool to identify new cryptic RNA pol II promoters and for improving the annotation of plant genomes. A first analysis conducted in Arabidopsis by using a compilation of associations of telo boxes with at least two site II motifs or a TEF box and a BLAST search with the sequences located downstream from these associations in the "A. thaliana GB experimental cDNA/EST (DNA) dataset" allowed us to identify new transcript units. This is illustrated in Figure 7 showing the identification in four intergenic regions and four introns of new transcripts which are not annotated in the TAIR database.

## Discussion

**Figure 8:**
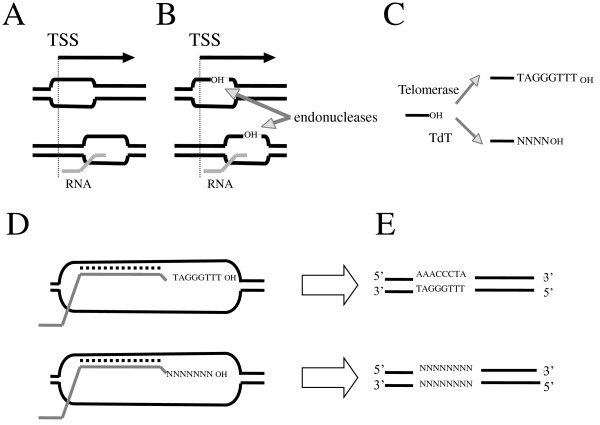
**Possible transcription-associated recombination mechanism**. A possible transcription-associated recombination mechanism is proposed for spreading of *telo *boxes, microsatellites and Y patches within plant genomes. **(A) **open transcription pre-initiation complex and R-loop at promoter-proximal pausing sites; **(B) **generation of free 3'OH recombinogenic ssDNA by endonucleases; **(C) **the free 3'OH ends are substrates for telomerase or terminal transferase; **(D) **3' end invasion at homologous open sites followed by error-prone DNA repair; **(E) **acquisition of a telomere repeat unit or new nucleotides. See text for comments. TSS: transcription start site. TdT: terminal transferase.

One remarkable item of data resulting from this study is the striking similarity observed in the genomic distribution of *telo *boxes and microsatellites. Their preferential location in 5' flanking regions can be assigned to their role in gene expression as has been reported for both *telo *boxes [11,12] and microsatellites [28,29]. However, we think that this preferential distribution in 5' regions could also reflect a common process involved in the acquisition of these motifs. We previously proposed a model involving the telomerase and recombination events to explain the spreading of *telo *boxes within *Arabidopsis *genome [9]. A schematic representation of this model and of its possible analogy with the acquisition process of microsatellites is shown in Figure 8. It can be summarized as follows: (i) Promoter regions are hot spots for recombination and it is well established that there is a relationship between recombination and chromatin accessibility to nucleases occurring during transcription initiation and elongation processes [30-32], (Figure 8A). (ii) Free 3'OH recombinogenic ssDNA is thus generated, (Figure 8B). (iii) These free 3'OH ends are potential substrates for telomerase which, in the absence of telomere repeats interacting with the telomerase anchor site, could act in a non-processive manner by adding only one telomere motif at the 3' end [33], (Figure 8C). It must be emphasized that, as for *rp *genes, there is also a strong correlation between cell cycle progression and telomerase expression in *Arabidopsis *[34]. (iv): The 3' end invasion at homologous open sites (Figure 8D) followed by error-prone DNA repair leads to the acquisition of a telomere repeat unit (Figure 8E). A related process has been suggested for the spreading of microsatellites in the human genome by 3'OH-extension of retrotranscripts [35]. As we suggested for the putative generation of *telo *boxes driven by the telomerase RNA template, the authors speculate that RNA guides could give rise to specific microsatellite sequences. In a similar manner, the spreading of simple repeated sequences such as Y patches could be achieved by addition of nucleotides to free 3' ends by a terminal transferase (TdT), (Figure 8D and 8E). The occurrence in angiosperms of a TdT activity has been reported in germinating wheat embryos [36]. During V(D)J recombination in mammals, the TdT contribute greatly to the generation of diversity in the immune repertoire and the addition of template-independent nucleotides frequently consists of purine or pyrimidine tracts [37]. The common feature in the hypothetical transcription-associated recombination processes mentioned above is the availability of a free 3' end for TdT, telomerase or other related hypothetical specific RNA-guided reverse transcriptase followed by error-prone DNA repair. In the context discussed here it is interesting to mention that similarly to our data showing a high frequency of *telo *boxes within 5' UTRs of genes encoding components involved in the biogenesis of ribosomes, 46.5% of translation-related genes in rice contain some microsatellites in their predicted 5' UTRs, (GCC/GGC)n contributing for about half of them [19 and our unpublished data].

Biogenesis of ribosomes is a crucial process requiring the coordinate expression of hundreds of genes. In the yeast *Saccharomyces cerevisiae *this synchronized expression is primarily accomplished at the transcriptional level and mediated through common upstream activating sequences including in most cases Rap1p binding sites (rpg boxes) and, in a small subset of rp genes, Abf1p binding sites [38,39]. In higher eukaryotes little is known about the transcriptional network controlling this regulon [40]. Studies conducted in our group over the last two decades have led to the identification of several transcriptional trans and cis-acting elements which participate in the over-expression of translational factor and rp genes in dividing plant cells [3,11,12,14,41]. The data reported in the present work suggest that the occurrence of telo boxes in the 5' flanking regions of rp genes is the rule not only in Arabidopsis but in angiosperms in general and therefore extend this observation to genes involved in the maturation of pre-rRNA. In agreement with data coming from a genome-wide analysis suggesting that the sequences AAACCCTA and TAGGGTTT are Arabidopsis core promoter elements [22], the majority of telo boxes observed in 5' flanking regions of plant translation-related genes are located within a narrow window located -50 to +50 relative to the transcription start site (TSS). The conservation of a topological association between telo boxes and site II motifs or TEF box cis-acting elements provides insights into the transcriptional regulation process required for the coordinate expression of plant genes involved in ribosome biogenesis. For several aspects, a parallel can be drawn between the putative role of telo boxes in plants and those achieved by the rpg cis-acting element in the yeast *S. cerevisiae*: (i) the rpg boxes (ACACCCAYACAY) show an homology with yeast telomere repeats (C_(1-3)_A)n and are both targets for the Rap1p pleiotropic protein involved in telomere metabolism and gene expression [42]; (ii) a common characteristic of yeast genes under the control of rpg boxes is their very high transcription rate during exponential growth. Up to now, the effect of telo boxes on expression was only observed in exponentially-growing cell cultures or in cycling cells of root primordia and young leaves [11-13]; (iii) among the yeast genes up-regulated in an rpg-dependent manner during exponential growth, genes involved in the biogenesis of ribosomes constitute a major class [38,43,44]; (iv) the interaction of Rap1p with the rpg box does not directly act as transcriptional activator but instead as a synergistic element that allows the activation by other regulatory proteins in participating in their recruitment in protein-protein interactions or in destabilizing the DNA duplex [38,45,46]. Similarly, in gain-of-function experiments, the telo box is not able by itself to activate gene expression in transgenic plants but acts in synergy with other cis-acting elements like site II motifs or TEF boxes [11,12]. Taken together, these observations support the hypothesis that there are functional similarities between the roles played by interstitial telomere motifs in plant promoters and those of the rpg box in yeast. We have estimated at about 10% the number of Arabidopsis genes harbouring a telo box within their 5' flanking regions suggesting that this element plays a much more general role than solely in the ribosome biogenesis. An intriguing question which might consequently be addressed concerns the meaning of the involvement in both yeast and angiosperms of interstitial telomere motifs in the expression of a set of genes whose expression is, at least for translation-related genes, correlated to cellular proliferation.

In contrast to that observed in vertebrates, many plant snoRNA genes are found in polycistronic clusters composed of homologous or heterologous snoRNAs [47]. Intronic snoRNA genes are frequently found in the genome of rice [26,27] whereas they are the exception in *Arabidopsis *[48]. There is currently little information on how the expression of plant snoRNA genes is coordinated with the expression of other components involved in the biogenesis of the translational apparatus. When nested within introns of genes involved in ribosome biogenesis such as fibrillarin SnRNP genes in *Arabidopsis *or several *rp *genes in *O. sativa *the co-expression process appears to be obvious. This co-expression process is much less clear when snoRNAs are expressed from independent promoters in non-intronic genes. Some plant non-intronic snoRNAs are RNA polymerase III products as suggested in *Arabidopsis *and rice by the characterization of dicistronic tRNA-snoRNA genes [47,49]. However, it remains to assess the proportion of non-intronic snoRNAs that are transcribed by pol III in plants. Our data suggest that, at least in *Arabidopsis*, this is probably the exception rather than the rule. The remarkable conservation of the topological association of *telo *boxes with site II motifs or TEF boxes observed in promoters of genes encoding ribosomal proteins or proteins required for pre-rRNA processing as well as within sequences found upstream of non-intronic snoRNA genes, strongly suggests that the association of these *cis*-acting elements and their interaction with related *trans*-acting factors might play a fundamental role in their coordinated transcription by RNA pol II. Moreover, we took advantage of the availability of TIGR-CERES data on the sequencing of full length *Arabidopsis *cDNAs to map the 5' end of several snoRNA precursors (Additional Files 3 and 4). These full-length cognate cDNAs were obtained by the "cap-trapping" method indicating that the identified RNA precursor molecules harbouring snoRNAs are indeed capped and polyadenylated RNA pol II transcripts. Once again, and as for *rp *genes, a parallel can be drawn between the putative role played by the *telo *box in plants and those achieved by the yeast rpg box in snoRNA gene expression. In *S. cerevisiae *the promoters of non-intronic snoRNA genes contain rpg boxes which are required for their full expression [50]. Thus, the analysis of conserved associations of *telo *boxes with site II motifs or TEF boxes allowed us to characterize new RNA pol II promoters involved in the biosynthesis of snoRNA precursors. A first analysis suggest that such an approach could be generalized to identify unexpected cryptic RNA pol II promoters within plant genomes (Figure 7). It would be of interest to investigate to what extent such promoters participate in the activation of expression in meristematic cycling cells, as is the case for plant *rp *or pre-rRNA processing genes showing a similar promoter configuration.

## Conclusion

The data reported in this work support the model previously proposed for the way *telo *boxes spread within plant genomes and provide new insights into a putative process for the acquisition of microsatellites in plants. The conserved topological association of *telo *boxes with site II or TEF1 *cis*-acting elements appears to be an essential feature of plant genes involved in the biogenesis of ribosomes and clearly indicates that most plant snoRNAs are RNA pol II products. This conserved association could provide a powerful tool to improve genome annotation in characterizing new cryptic RNA pol II promoters.

## Methods

### Sequence data sources

Analysis of *Arabidopsis *sequences was carried out using the TAIR9 datasets http://www.arabidopsis.org. The analysis conducted by using the TAIR9 5'UTR (DNA) and the TAIR9 3' UTR (DNA) datasets does not include the sequences of putative introns within the 5' or 3' flanking non coding regions. The *Arabidopsis *rRNA processing protein and snoRNA genes were obtained from TAIR.

The *O. sativa *genome annotation data version 5 was downloaded from the Rice Genome Annotation Project database http://rice.plantbiology.msu.edu/. The "all.UTR" file containing the UTR sequences for 34793 gene models of the 12 pseudomolecules was used. The sequence of 5' flanking regions of rice ribosomal protein gene were extracted from the Ribosomal Protein Gene database http://ribosome.miyazaki-med.ac.jp/. The list of putative rice snoRNA and accession numbers were obtained from the literature [27]. For each rice snoRNA, we extracted the Genbank sequence by using its accession number. All the snoRNA were searched for in the complete genomic sequence of *Oryza sotiva *by using NCBI Blastn with default parameters. Some of the clusters of snoRNA were obtained from the NCBI nucleotides database and were used to assign snoRNA to clusters. Others were assigned by using their chromosomic location and their positions on the chromosome. 60 clusters (instead of 68 given in Chen et al. [27]) were assigned to chromosomic loci thanks to the list of snoRNA given for each cluster. We also proposed some new clusters. For clusters 35, 36 and 37, it was not possible to assign snoRNA to clusters precisely. Nor was it possible to assign each sequence to a chromosomic region in the complete sequence of *Oryza sotiva*. Indeed, for some of the snoRNA we did not find significant similarities to anything in the entire genome of *Oryza. sativa*.

### Motifs search

The command line version of the PatMatch software [51] was used to scan the different compartments of the genome for the presence of several nucleotide patterns: *telo *box (AAACCCTA) and 6 associated permutations of the *telo *box motif (AACCCTAA, ACCCTAAA, CCCTAAAC, CCTAAACC, CTAAACCC and TAAACCCT); a control sequence (AAACCTCA), and 6 associated permutations (AACCTCAA, ACCTCAAA, CCTCAAAC, CTCAAACC, TCAAACCT and CAAACCTC); the site II motifs (TGGGCY); the TEF1 box (ARGGRYNNNNNGYA); the (GCC)_6 _and (GAA)_6 _microsatellite motifs; and the (Y)18 pyrimidine block.

For protein coding genes, a region of 500 nt was scanned upstream of the translation initiation codon. In the case of snoRNA genes, for each cluster found in an ORF, a region of 1000 nt was extracted in the 5' region before the ATG of the host gene. For each cluster found in an intergenic region, 1000 nt were extracted before the beginning of the first snoRNA of the cluster. For individual snoRNA, a region of 1000 nt was extracted just before the beginning of the 5' region of the mature snoRNA.

### Chi-square analysis

The expected frequency of telo-box motif in each genome compartment under the assumption of a uniform distribution in the genome was determined as the ratio of each compartment size to the genome size. For each compartment, a chi-square test was performed between observed and expected counts of telo-box motif as compared to observed and expected counts in the rest of the genome. A combined chi-square test was performed as the sum over compartments of the square of the difference between observed and expected counts divided by expected count.

### Mapping of cDNA

Putative transcripts located downstream of associations of *telo *boxes with site II motifs or TEF1 boxes were characterized by using sequences located downstream of these associations, Blastn and *A. thaliana *GB experimental cDNA/EST or Green Plant GB experimental cDNA/EST datasets.

## Authors' contributions

BL designed the study, realized all the analysis on *A. thaliana *and wrote the manuscript. JFR contributed to search for motifs and their statistical analysis in *O. sativa*. CG contributed to search for snoRNA and the analysis of their 5' flanking region in *O. sativa*. All authors contributed to editing of the manuscript. All authors read and approved the final manuscript.

## Supplementary Material

Additional File 1 **This file contains a table showing in *O. sativa *the location of *telo *boxes, site II motifs, TEF1 boxes and transcription start sites (TSS) relative to the translation initiation codon of ribosomal protein genes**. Click here for file

Additional File 2 **This file contains a table (table **2A**) showing in *A. thaliana *the location of *telo *boxes, site II motifs, TEF1 boxes and transcription start sites relative to the translation initiation codon of genes annotated in TAIR as encoding protein involved in rRNA processing**. In table 2B is shown the occurrence of *telo *boxes in *O. sativa *orthologous genes. Click here for file

Additional File 3 **This file contains a table showing in *A. thaliana *the location of *telo *boxes, site II motifs, TEF1 boxes and transcription start sites of snoRNA precursors relative to the 5' end of the first mature snoRNA in independent clusters, the 5' end of the mature orphan snoRNA or relative to the translation initiation codon when snoRNA genes are nested within a protein coding gene**. Click here for file

Additional File 4 **This file contains a table showing in *O. sativa *the location of *telo *boxes, site II motifs, TEF1 boxes and transcription start sites of snoRNA precursors relative to the 5' end of the first mature snoRNA in independent clusters, the 5' end of the mature orphan snoRNA or relative to the translation initiation codon when snoRNA genes are nested within a protein coding gene**. Click here for file

Additional File 5 **This file contains a table giving the chromosomic location and positions of snoRNAs**. Click here for file
