# Supplementary material for: Distribution of short interstitial telomere motifs in two plant genomes: putative origin and function
Source: BMC Plant Biol. 2010 Dec 20;10:283. doi: 10.1186/1471-2229-10-283 (PMC3022908; doi:10.1186/1471-2229-10-283)
Supplement: Additional File 1 — This file contains a table showing in O. sativa the location of telo boxes, site II motifs, TEF1 boxes and transcription start sites (TSS) relative to the translation initiation codon of ribosomal protein genes. [file 1471-2229-10-283-S1.PDF]

Additional File 1

O. sativa ribosomal protein genes. Location of *telo* boxes, site II motifs, TEF1 boxes and TSS relative to the translation initiation codon.

Locus	product	telobox	site II	5' end mRNA
Os07g10660	rpS2-1	-127	-148,-160,-170,-184,-206,-236,-258,-460	-
Os03g59310	rpS2-2	-22,-127	-160,-165,-176,-207,-222,-265,-316,-420	-77
Os07g23800	rpS2-3	-292	-87,-674	-
Os03g34040	rpS2-4	-	-153,-606	-
Os03g38000	rpS3-1	-75,-176	-219,-241,-248,-274,-502	-113
Os07g41750	rpS3-2	-61,-233	-	-
Os03g10340	rpS3A-1	-64	-185,-209,-240	
Os12g21800	rpS3A-2	-104	-171	-
Os02g18550	rpS3A-3	-25,-374	-165,-256,-310,-347	-
Os02g01560	rpS4-1	-139	-160 (TEF),-188,-346,-396,-398,-423,-514	-103
Os01g25610	rpS4-2	-150	-164,-174,-206,-230	-98
Os05g30530	rpS4-3	-371	-174	-
Os11g29190	rpS5-1	-77	-155,-174	-109
Os01g01060	rpS5-2	-37,-172	-129,-135,-147	-
Os07g42950	rpS6-1	-77,-126	-156,-223,-241,-265,-294,-322,-392	-
Os03g27260	rpS6-2	-26	-176	-49
Os03g18580	rpS7-1	-240	-262,-478	-
Os03g18570	rpS7-2	-209,-253	-275,-290,-321,-340	-
Os05g27940	rpS7-3	-824	-868,-878,-895,-901	-822
Os04g28180	rpS8-1	-107,-148	-120,-256,-292,-299	-102
Os02g28810	rpS8-2	-129	-145,-175	-
Os11g38940	rpS9-1	-128	-155,-171,-198,-221	-62
Os11g38960	rpS9-2	-112	-139,-155,-182,-205,-222	-64
Os03g05980	rpS9-4	-	-7,-28	-
Os11g38940	rpS9-5	-128	-155,-171,-198,-221	-62
Os07g43510	rpS9-6	-861	-	-
Os04g56670	rpS9-7	-196	-239,-248,-257,-281	-224
Os10g38230	rpS9-8	-	-	-367
Os01g73160	rpS10-1	-91	-134,-147,-175	-90
Os02g34460	rpS10-2	-69	-258,-282,-284	-
Os04g35090	rpS10-3	-96,-146	-183,-194,-216	-99
Os07g37780	rpS11-1	-99	-188,-198,-444	-
Os04g52350	rpS11-2	-99	-188,-198,-432	-129
Os07g37770	rpS11-3	-126	-160,-175,-185	-126
Os04g52360	rpS11-4	-126	-160,-175,-185	-90
Os04g52350	rpS11-5	-99	-188,-198,-432	-129
Os08g10610	rpS11-6	-70	-	-
Os04g42380	rpS11-7	-91	-158,-168,-207,-561	-84
Os07g12650	rpS12-1	-181	-267,-288	-211
Os07g05580	rpS12-2	-167	-249,-270,-302,-313,-341,-347,-359	-196
Os08g02410	rpS13-1	-99	-168,-191,-213,-515	-91
Os08g02400	rpS13-2	-137	-245,-652	-104
Os07g38540	rpS13-3	-	-2,-143,-164,-182,-277	-94
Os02g45490	rpS13-4	-	-	-
Os02g06700	rpS14-1	-147	-210,-216,-245,-255,-276,-300	-94
Os04g33750	rpS14-2	-56	-123,-154	-86
Os02g33140	rpS14-3	-64	-133,-147,-167,-398	-81
Os02g01540	rpS14-4	-	-68,-280,-289	-
Os03g58430	rpS15-1	-92	-162,-169,-198,-249	-99
Os07g08660	rpS15-2	-	-618	-
Os07g10720	rpS15A-1	-1098	-358,-1183,-1218,-1240	-1105
Os02g27760	rpS15A-2	-	-	-
Os02g15610	rpS15A-3	-174	-186	-89
Os01g06000	rpS15A-4	-	-	-
Os11g03400	rpS16-1	-116	-198,-216,-236,-642	-41
Os12g03090	rpS16-2	-121,-262	-138,-159,-169,-174,-189,-201	-82

Os03g55930	rpS16-3	-	-	-63
Os10g27190	rpS17-1	-149,-627	-227,-236,-251,-262,-994	-98
Os03g01900	rpS17-2	-116	-198,-216,-238,-642	-38
Os07g07700	rpS18-1	-90	-197,-209,-248,-590	-100
Os03g58050	rpS18-2	-136	-178,-184,-219,-221,-329,-1052	-92
Os07g07720	rpS18-3	-	-251,-756	-
Os07g07770	rpS18-4	-	-	-
Os03g49710	rpS18-5	-	-157,-174	-109
Os03g31130	rpS19-1	-163	-186,-206,-216,-949	-67
Os03g31090	rpS19-2	-81	-211,-231,-241,-422,-821	-90
Os03g14530	rpS20-1	-59	-117,-152	-61
Os10g08930	rpS20-2	-86	-145,-152,-177	-105
Os06g04290	rpS20-3	-31	-251,-253,-284	-
Os03g10060	rpS20-4	-	-	-
Os03g22460	rpS21-1	-645,-1346	-666,-681,-700,-724,-933	-597
Os03g46490	rpS21-2	-	-	-
Os03g60400	rpS23-1	-	-240	-80
Os01g61820	rpS23-2	-154	-167,-206,-220	-
Os10g20990	rpS23-3	-	-380	-
Os10g20910	rpS23-4	-	-	-
Os05g13660	rpS23-5	-	-	-
Os02g13530	rpS24-1	-141	-158,-184,-198,-216,-232	-94
Os01g52490	rpS24-2	-89	-127,-180,-207,-216,-257	-
Os06g36160	rpS24-3	-134	-171,-193,-221,-518,-520	-
Os08g44480	rpS25-1	-102	-656,-668,-685,-930,-949,-966,-973	-
Os09g39540	rpS25-2	-110,-400	-617	-61
Os11g05560	rpS25-3	-668	-76,-108,-156,-170	-
Os01g60790	rpS26-1	-78	-197,-987	-
Os05g39960	rpS26-2	-48	-120,-129	-51
Os03g60160	rpS26-3	-85	-216,-225,-235,-251,-287,-648	-
Os04g27860	rpS27-1	-	-	-
Os04g32710	rpS27-2	-101	-205,-229,-243,-258	-99
Os01g22490	rpS27A-1	-101	-206,-218,-237	-109
Os05g06770	rpS27A-2	-67	-122,-179,-211	-
Os03g07100	rpS28	-	-332	-
Os11g41610	rpS29-1	-137	-165,-176,-190,-216	-87
Os03g56240	rpS29-2	-27	-108,-134,-157,-165	-53
Os12g32380	rpS29-3	-54,-135	-161,-174,-200,-212	-
Os06g07580	rpS30	-89	-155,-177,-191,-223,-240,-774,-940	-67
Os07g42450	rpSA-1	-156	-127,-137,-184,-521	-95
Os03g08440	rpSA-2	-140	-194,-200	-136
Os12g07010	rpL3-1	-290	-363,-402,-427,-476	-
Os11g06750	rpL3-2	-250	-358,-363,-378,-386	-
Os07g08330	rpL4-1	-30,-106	-188,-193,-215,-237,-271	-62
Os03g58200	rpL4-2	-116	-190,-195,-225	-52
Os01g67140	rpL5-1	-85	-239,-253	-55
Os01g67130	rpL5-2	-85,-138	-236,-283	-59
Os04g39700	rpL6-1	-119	-147,-169	-91
Os02g37860	rpL6-2	-121	-135,-158,-489	-
Os08g13690	rpL7-1	-59,-180	-332,-356,-384,-398	-151
Os04g51630	rpL7-2	-215	-269,-309,-325,-988	-
Os08g42920	rpL7-3	-72	-135,-158,-176,-189,-198,-223	-84
Os08g23710	rpL7A-1	-209	-231,-256,-520,-522	-92
Os01g70010	rpL7A-2	-49	-627	-
Os06g16290	rpL7A-3	-94	-203,-209,-224	-
Os02g49610	rpL7A-4	-184	-131	-
Os10g03540	rpL7A-5	-81	-129,-154,-160,-181	-70
Os03g13800	rpL7A-6	-124	-277	-121
Os12g38000	rpL8	-62	-171,-216,-226,-245,-545,-547,-564	-65
Os09g31180	rpL9-1	-132	-100,-232,-259,-278,-318	-79
Os02g01330	rpL9-2	-164	-182,-200,-222,-224,-266	-
Os11g11390	rpL10-1	-74	-140,-146,-177,-179,-193	-70
Os05g07700	rpL10-2	-56	-150,-161,-171,-184	-62
Os03g21470	rpL10-3	-	-265,-282,-302	-
Os08g44380	rpL10A-1	-171	-309,-318,-354,-369	-231
Os08g44450	rpL10A-2	-89,-489	-163,-180,-197	-70
Os02g21660	rpL10A-3	-171,-232	-284,-291,-861	-178

Os01g64090	rpL10A-4	-189	-235,-240,-262	-
Os08g44450	rpL10A-5	-89,-489	-163,-180,-197	-70
Os08g44450	rpL10A-6	-1108	-1182,-1199,-1216	-417
Os06g35470	rpL10A-7	-	-122,-144,-178,-211,-223	-
Os05g11710	rpL11-1	-73	-199,-221,-237,-403	-74
Os01g10820	rpL11-2	-111	-152 (TEF), -486	-88
Os06g35730	rpL11-3	-	-193	-
Os02g14050	rpL11-4	-83	-120,-122,-167,-195,-204	-103
Os02g14070	rpL11-5	-	-165,-212,-296	-
Os11g31020	rpL11-6	-16	-238	-
Os06g50290	rpL11-7	-16	-171,-237	-
Os02g47140	rpL12-1	-83,-139	-178,-203,-230,-248	-97
Os04g50990	rpL12-2	-84	-156,-161,-234,-607	-
Os06g02510	rpL13-1	-476	-542,-553,-559,-590,-604	-
Os03g37970	rpL13-2	-362	-453,-503	-340
Os03g54890	rpL13A-1	-100	-155,-167,-191	-85
Os07g01870	rpL13A-2	-90	-147,-167,-183	-
Os03g54860	rpL13A-3	-46	-156,-168,-198	-
Os11g23880	rpL13A-4	-410	-441,-456	-
Os01g54540	rpL13A-5	-274,-458	-175,-192,-202	-117
Os02g40880	rpL14-1	-69	-213,-227,-247,-253	-
Os04g43540	rpL14-2	-	-227,-235,-263,-298	-
Os03g40180	rpL15-1	-66,-140	-200,-211,-248,-899	-45
Os05g19370	rpL15-2	-54	-135,-146,-179,-472,-480,-725,-797	-65
Os06g20360	rpL15-3	-	-	-
Os09g08430	rpL17-1	-135	-144,-156,-172,-197	-79
Os08g41810	rpL17-2	-116	-146,-164,-166,-223,-259	-125
Os07g47780	rpL18-1	-51	-126,-191,-215	-58
Os03g22180	rpL18-2	-40,-839	-131	-49
Os07g47780	rpL18-3	-51	-126,-191,-215	-55
Os05g06310	rpL18-4	-130	-146,-155,-189,-199,-221,-325,-713	-75
Os01g54870	rpL18A-1	-147	-176,-195,-203	-
Os01g47660	rpL18A-2	-16,-132	-140,-161,-185	-117
Os05g49030	rpL18A-3	-	-103	-
Os03g21940	rpL19-1	-148	-170,-175,-184,-697	-95
Os03g38260	rpL19-2	-102,-791	-131,-138,-140,-432,-434,-564,-890	-44
Os07g47430	rpL19-3	-	-	-
Os03g04750	rpL21-1	-76	-121,-155,-181,-183	-
Os10g32820	rpL21-2	-85	-167,-172,-185,-235	-85
Os07g47710	rpL22-1	-69	-123,-165,-219,-253,-758	-77
Os03g22340	rpL22-2	-76	-193,-235,-248	-85
Os10g32920	rpL23-1	-	-90,-98,-105,-126,-158	-46
Os03g04590	rpL23-2	-66	90 (TEF),-249	-
Os02g56960	rpL23-3	-	-433	-37
Os01g57950	rpL23-4	-202	-	-
Os05g22720	rpL23-5	-	-	-
Os12g10610	rpL23-6	-	-	-
Os12g42180	rpL23-7	-1271	-1373,-1477	-491
Os01g24690	rpL23A-1	-118,-136	-148,-191,-200,-220	-
Os04g42270	rpL23A-2	-878,-941<br>-1625	-957,-990,-1642	-871
Os01g59990	rpL24-1	-1082	-1105,-1122,-1167,-1354	-1059
Os05g40820	rpL24-2	-1377	-1411,-1453,-1474,-1509,-1518	-1355
Os07g12250	rpL24-3	-623	-778,-812,-1100,-1114	-643
Os01g33050	rpL24-4	-146	-263,-278,-295	-203
Os07g19190	rpL24-5	-195	-247,-258,-295,-370	-188
Os12g05430	rpL26-1	-32	-175,-228,-310,-312	-53
Os11g05370	rpL26-2	-32	-149,-181,-195,-221,-270	-11
Os01g04730	rpL26-3	-31	-221,-230,-259,-280	-73
Os10g41470	rpL27-1	-41,-104	-131 (TEF),-268,-306,-338	-54
Os02g18380	rpL27-2	-110,-159	-182 (TEF),-331,-352,-389	-
Os03g29460	rpL27A-1	-172	-203,-209,-237,-627	-30
Os07g42170	rpL27A-2	-88	-120,-128,-133	-50
Os02g07890	rpL27A-3	-164	-185,-190,-224,-237	-54
Os01g51020	rpL28-1	-1221	-1253,-1280,-1310,-1319	-1191
Os05g46430	rpL28-2	-614	-674,-692,-734,-744,-749,-769	-568
Os02g57540	rpL28-3	-614	-640,-650,-674,-680,-703	-

Os05g28750	rpL29-1	-189	-276,-289,-300	-
Os01g19840	rpL29-2	-185	-338,-354,-407	-
Os01g16890	rpL30-1	-97	-153 (TEF),-171,-177,-210	-90
Os05g41110	rpL30-2	-82,-257	-132 (TEF),-147,-163,-183,-722	-80
Os01g59730	rpL30-3	-	-	-
Os08g39500	rpL31-1	-62	-119,-142,-184	-42
Os02g48660	rpL31-2	-81	-150,-177	-80
Os06g21480	rpL31-3	-74	-182	-
Os08g41300	rpL32-1	-228	-314,-337,-360,-379,-397,-428,-441, -489,-485,-790	-188
Os09g32520	rpL32-2	-192	-233,-253,-284	-182
Os09g32530	rpL32-3	-158	-170,-184,-215,-269	-
Os09g32500	rpL32-4	-197	-209,-225,-252,-441,-816,-848,-856	-
Os09g24690	rpL34-1	-44	-180,-196,-211	-87
Os08g33920	rpL34-2	-22	-175,-208,-218,-272,-338	-74
Os08g06040	rpL34-3	-81	-	-74
Os04g30730	rpL35-1	-135	-154,-164,-200,-226	-69
Os02g30050	rpL35-2	-135	-158,-168,-176,-198,-200,-230	-56
Os06g51530	rpL35-3	-486,-501	-	-321
Os05g48220	rpL35A-1	-351	-204,-218,-233,-464	-94
Os05g48310	rpL35A-2	-129	-171,-201,-247,-270,-312,-391	-
Os02g54470	rpL35A-3	-	-420,-422,-714	-
Os05g38520	rpL36-1	-75,-162	-231,-240,-253,-646	-159
Os01g62350	rpL36-2	-181,-212	-267,-296,-332	-198
Os07g33880	rpL36A-1	-278	-388,-394,-432,-453	-
Os07g26740	rpL36A-2	-69	-140,-154,-441,-443,-484,-492	-98
Os07g33980	rpL36A-3	-275	-385,-391,-429,-450	-
Os07g33930	rpL36A-4	-275	-385,-391,-429,-450	-
Os07g33870	rpL36A-5	-30	-175,-196,-203	-64
Os07g34000	rpL36A-6	-31,-137	-166,-181,-187	-85
Os07g33950	rpL36A-7	-31,-137	-166,-181,-187	-85
Os07g33900	rpL36A-8	-31,-137	-166,-181,-187	-49
Os07g33860	rpL36A-9	-939	-	-
Os07g33970	rpL36A-10	-	-993	-
Os07g33920	rpL36A-11	-	-993	-
Os02g02130	rpL37-1	-22,-69	-111,-121,-149,-187,-207	-53
Os02g56990	rpL37-2	-	-100,-149,-185,-211	-81
Os08g03450	rpL37-3	-22,-78	-120,-130,-158	-67
Os05g48320	rpL37A-1	-121	-164,-196,-229,-293,-348	-81
Os01g48770	rpL37A-2	-119	-151,-165,-192,-206,-233	-81
Os11g24610	rpL38-1	-102	-141,-143,-161,-179,-188,205,-223, -232,-283	-
Os07g36250	rpL38-2	-97,-303	-153,-170,-193	-
Os05g43160	rpL38-3	-	-677	-
Os06g08320	rpL39-1	-17,-657	-85,-94,-103,-114,-192,-209	-45
Os02g55390	rpL39-2	-39	-113,-127,-155,-189,-195,-212,-245, -270	-
Os02g55370	rpL39-3	-30	-84,-98,-127,-160,-241,-667,-913	-41
Os09g39500	rpL40-1	-146,-871	-158,-193,-511,-545	-106
Os03g13170	rpL40-2	-127	-208,-231	-
Os09g31010	rpL40-3	-	-250,-271,-281,-299	-44
Os07g30640	rpL40-4	-128,-148	-251 (TEF),-548	-59
Os03g15370	rpL40-5	-	-	-
Os09g27930	rpL40-6	-	-246,-264,-274,-991	-
Os09g31030	rpL40-7	-	-306,-337,-356	-78
Os08g03640	rpLP0-1	-127,-213	-194,-203,-247,-257,-273	-130
Os12g03880	rpLP0-2	-	-146,-174,-187	-
Os11g04070	rpLP0-3	-	-167,-195,-208,-357,-377,-435	-
Os08g02340	rpLP1	-185,-250	-265,-277,-373,-388,-418,-497,-534	-193
Os05g37330	rpLP2-1	-846,-930, -1524	-978,-985,-991,-1073,-1102,-1117, -1131	-854
Os01g09510	rpLP2-2	-768	-936,-951,-971,-993,-1002	-788
Os08g15180	rpLP2-3	-	-280,-296,-904,-991,-1038,-1087, -1224	-
Os02g32760	rpLP2-4	-1283	-1354	-1295
Os07g28710	rpLP2-5	-	-	-
Os07g14750	rpLP2-6	-937	-	-
