# Supplementary material for: Distribution of short interstitial telomere motifs in two plant genomes: putative origin and function
Source: BMC Plant Biol. 2010 Dec 20;10:283. doi: 10.1186/1471-2229-10-283 (PMC3022908; doi:10.1186/1471-2229-10-283)
Supplement: Additional File 2 — This file contains a table (table 2A) showing in A. thaliana the location of telo boxes, site II motifs, TEF1 boxes and transcription start sites relative to the translation initiation codon of genes annotated in TAIR as encoding protein involved in rRNA processing. In table 2B is shown the occurrence of telo boxes in O. sativa orthologous genes. [file 1471-2229-10-283-S2.PDF]

Additional File 2

A

Arabidopsis rRNA processing protein coding genes. Location of *telo* boxes, site II motifs, TEF1 boxes and TSS relative to the translation initiation codon.

Locus	Product	*telo* boxes	Site II motifs or TEF boxes	5' end mRNA
At1g07840	leucine zipper factor	-	-608	-549
At1g48920	Nucleolin	-60	-139 (TEF),-341	-91
At1g56110	Nop56p	-20, -68	-128, -134	-58
At1g63780	Small nucleolar ribonucleoprotein	-38	-166,-186,-198	-124
At2g20490	Nop10p	-146	-232,-270,-288	-175
At2g37990	ribosome biogenesis regulatory protein (RRS1)	-58	-	-78
At2g40360	transducin family protein	-74,-162	-108,-195	-55
At2g43190	ribonuclease MRP complex	-288	-	-235
At2g43650	EMB2777 (EMBRYO DEFECTIVE 2777)	-65,-205	-232,-244,-264	-192
At2g47990	transducin family nucleolar protein	-68	-134,-156	-76
At3g03920	Gar1 H/ACA rnp complex subunit 1	-68,-120	-326,-348	-101
At3g05060	SAR DNA-binding protein	-144,-386,-483	-507	-402
At3g09720	RNA helicase involved in rRNA processing	-352	-480,-492	-350
At3g11964	pre-rRNA processing protein	-58,-88	-187,-218	-
At3g12860	Nop56	-18,-99	-160,-182,-196	-108
At3g21540	transducin family protein	-62	-	-
At3g22660	rRNA processing protein EBP2	-103,-220,-317	-	-390
At3g28230	Small subunit processome	-100,-140	-166,-180,-200	-99
At3g47420	dimethyladenosine transferase	-64	-	-89
At3g57000	Nucleolar protein	-297,-414	-401,-429	-342
At3g57150	Pseudouridine synthase	-13,-97,-106	-159,-190	-57
At3g60360	U3 snrp 11	-111,-239,-367	-445 (TEF)	-398
At4g02400	U3 ribonucleoprotein	-44	-	-54
At4g04940	transducin/WD-40 repeat protein	-141	-	-141
At4g05410	transducin/WD-40 repeat protein	-21	-194,-223,-260	-106
At4g21130	similar to man and yeast U3-55K genes	-45	-174	-11
At4g22380	Ribosomal protein L7Ae	-33	-	-78
At4g25630	Fibrillarin 2 (AtFib2)	-117	-451,-467,-488, -525	-105
At4g25730	FtsJ-like methyltransferase	-13,-84	-160,-180	-62
At4g26600	methyltransferase Nop2p	-18	-116,-139,-162	-62
At4g28450	SOF1-like protein	-127	-170,-201,-227	-157
At4g34730	ribosome-binding factor A	-	-	-2403
At5g08180	H/ACA rnp complex subunit 2	-62,-96,-142	-157,-193,-205	-81
At5g08420	RNA binding protein	-135	-218,-253,-268	-142
At5g08600	U3 ribonucleoprotein	-49	-	-31
At5g15750	RNA-binding S4 protein	-36,-269,-301	-424,-435	-346
At5g18180	H/ACA ribonucleoprotein	-50,-146	-	-
At5g20160	Ribosomal protein L7Ae	-16,-33	-136,-160	-99
At5g20600	Nop52	-103	-142 (TEF)	-90
At5g25730	FtsJ methyltransferase	-13,-84	-181	-62
At5g27120	SAR DNA-binding protein	-370,-462	-506,-523	-362
At5g46420	rRNA processing protein RimM	-	-	-44
At5g51280	DEAD-box protein	-100	-253,-276	-221
At5g52470	Fibrillarin 1	-130	-285,-300	-103
At5g52490	Fibrillarin 3	-109	-	-
At5g61330	rRNA processing protein EBP2	-955	-	-918
At5g66360	rRNA adenine dimethylase	-	-116,-153,-160	-100
At5g66540	U3 small nucleolar ribonucleoprotein	-28	-182,-194,-216	-117
At5g67630	DNA helicase	+132	-130,-142	-24

B

Orthologous *O. sativa* rRNA processing coding genes. Location of *telo* boxes, site II motifs, TEF1 boxes relative to the translation initiation codon.

**Locus**	**Arabidopsis orthologous gene**	**product**	***telo* boxes**	**Site II motifs or TEF boxes**
Os04g52960	At1g48920	Nucleolin	-127	-
Os03g22880	At1g56110	Nop56p	-62	-919,-981
Os08g05880	At1g63780	Small nucleoprotein	-25, -58	-139,-149,-165,-181,-188
Os07g25440	At2g40360	transducin	-	-231,-260,-301,-531,-604
Os07g01510	At2g43650	EMB 2777	-53	-110,-133,-159,-376
Os07g12320	At2g47990	transducin	-29	-94,-931
Os04g58830	At2g37990	transducin	-	-118, -142,-341
Os11g37080	At3g03920	Gar1 H/ACA rnp complex subunit 1	-45,-105,-441	-166
Os03g22740	At3g05060	SAR DNA-binding protein	-306	-320,-366,-403,-471
Os07g45360	At3g05060	RNA helicase	-48	-533,-610
Os07g10350	At3g11964	Pre-rRNA processing protein	-	-
Os07g46720	At3g12860	Nop56p	-47	-271
Os03g05720	At3g21540	transducin	-54	-
Os01g01510	At3g28230	Small subunit processome	-53	-110,-133,-159,-376
Os04g46880	At3g47420	dimethyladenosine transferase	-	-
Os02g18830	At3g57000	Nucleolar protein	-74,-244	-
Os07g44190	At3g57150	Pseudouridine synthase	-26	-137,-139,-180,-185,-192
Os01g59500	At3g60360	U3 snrp 11	-177	-191,-228,-233,-257,-267
Os03g22320	At4g02400	U3 ribonucleoprotein	-	-141,-146,-189
Os03g42770	At4g05410	transducin	-226	-261,-271
Os02g57590	At4g25630	Fibrillarin 2	-64,-82,-134	-190 (TEF)
Os05g49230	At4g25730	methyltransferase	-106	-182,-192,-213,-219,-252, -258,-340
Os02g49270	At4g26600	Nop2p	-	-63,-112,-123,-159
Os01g13730	At4g28450	SOF1 like protein	-126	-254,-268,-281,-906
Os01g16290	At5g08180	H/ACA rnp complex subunit 2	-94	-203,-209,-224
Os10g31520	At5g08420	RNA binding protein	-135	-112,-172,-192,-220
Os03g22320	At5g08600	U3 ribonucleoprotein	-714	-141,-146,-189
Os04g56670	At5g15750	RNA binding protein	-196	-239,-248,-257,-281
Os11g37080	At5g18180	H/ACA ribonucleoprotein	-45,-105,-442	-166
Os03g13800	At5g20160	Ribosomal protein L7Ae	-124	-
Os04g56350	At5g20600	Nop52p	-28	-254,-288,-321,-779,-823
Os03g22740	At5g27120	SAR DNA-binding protein	-306	-320,-366,-403,-471
Os02g05660	At5g51280	DEAD-box protein	-10	-174,-196,-242
Os05g08360	At5g52470	Fibrillarin 1	-60,-79	-
Os01g34200	At5g61330	rRNA adenine dimethylase	-	-124,-160,-171
Os12g04010	At5g66540	U3 snrnp	-103	-
Os06g08770	At5g67630	DNA helicase	-96	-204,-219,-436
