# Supplementary material for: Distribution of short interstitial telomere motifs in two plant genomes: putative origin and function
Source: BMC Plant Biol. 2010 Dec 20;10:283. doi: 10.1186/1471-2229-10-283 (PMC3022908; doi:10.1186/1471-2229-10-283)
Supplement: Additional File 3 — This file contains a table showing in A. thaliana the location of telo boxes, site II motifs, TEF1 boxes and transcription start sites of snoRNA precursors relative to the 5' end of the first mature snoRNA in independent clusters, the 5' end of the mature orphan snoRNA or relative to the translation initiation codon when snoRNA genes are nested within a protein coding gene. [file 1471-2229-10-283-S3.PDF]

## Additional File 3

Arabidopsis snoRNA genes. The location of telo boxes, site II motifs, TEF boxes and TSS is indicated relative to the 5' end of the mature snoRNA (first snoRNA when found in an independent cluster) or relative to the translation initiation codon of genes harbouring intron-encoded snoRNAs.

| *Locus* | **product** | ***telo* box** | *Site II or TEF* | **5' mRNA** |
|---|---|---|---|---|
| At1g03506<br>At1g03502 | snoRNA cluster | -240,-343 | -377 (TEF) | -310 |
| At1g03743<br>At1g03746 | snoRNA cluster | -140 | -181,-210,-239,-263 | - |
| At1g03935 | snoRNA | -274,-349 | -381 (TEF) | -301 |
| At1g07702 | snoRNA | - | - | - |
| At1g12013<br>At1g12015 | snoRNA cluster | -108 | -210,-218,-226,-244 | - |
| At1g19373<br>At1g19376 | snoRNA cluster | -180 | -264,-299 | - |
| At1g20015 | snoRNA | -160 | -208,-235 | EST |
| At1g26233<br>At1g26235 | snoRNA cluster | - | -354 | - |
| At1g32385 | snoRNA | -427 | - | -379 |
| At1g74453<br>At1g74456 | snoRNA cluster | -320 | -459,-481 | -342 |
| At1g75163<br>At1g75166 | snoRNA cluster | -400 | -442,-464 | -341 |
| At2g05765 | snoRNA | -616 | -666,-677,-755,-818 | -556 |
| At2g17295 | snoRNA | -587 | - | -539 |
| At2g20723<br>At2g20722<br>At2g20721 | snoRNA cluster | -302 | -354,-366,-405,-415 | -294 |
| At2g35382<br>At2g35384<br>At2g35387 | snoRNA cluster | -225 | -355 | -163 |
| At2g35742 | snoRNA | -138,330 | -625,-648,-670 | - |
| At2g35744<br>At2g35747 | snoRNA cluster | -314,-450 | -523,-605 | -408 |
| At2g43137<br>At2g43138<br>At2g43139<br>At2g43141 | snoRNA cluster, nested within a gene encoding an hypothetical protein | -282 from ATG | -296,-347 from ATG | -23 from ATG |
| At3g01313<br>At3g01316 | snoRNA cluster | -234 | -372,-440 | -176 |
| At3g03743<br>At3g03746 | snoRNA cluster | -140 | -181,-210,-239,-363 | - |
| At3g13525 | snoRNA | -84 | -155,-162,-177 | - |
| At3g14475 | snoRNA | - | - | - |
| At3g21805 | snoRNA | -43,-159 | -226,-271 | - |
| At3g24612<br>At3g24614<br>At3g24615 | snoRNA cluster | -87,-167 | -338 | -140 |
| At3g27865 | snoRNA | -73,-201 | -141,-154,-279 | -84 |
| At3g47342<br>At3g47347<br>At3g47348 | snoRNA cluster | -233,-327,-423 | -468,-502,-535 | -345 |
| At3g50825 | snoRNA | -213 | - | -161 |
| At3g58193<br>At3g58196 | snoRNA cluster | -207 | -282,-295,-339 | - |
| At4g02555 | snoRNA | -6,-224 | -276,-302 | - |
| At4g03295 | snoRNA | -322,-355,-432 | -535,-550,-578 | -398 |
| At5g05048 | snoRNA | -194 | -256,-282,-302 | - |
| At4g13245 | snoRNA | -429 | -516,-532 | EST |
| At4g15258 | snoRNA | -92,-194 | -241,-258 | -106 |
| At4g25631 | snoRNA, nested within fibrillarin gene | -117,-129 from ATG | -224 (TEF) from ATG | -106 from ATG |
| At4g26365 | snoRNA | -651 | -707,-727 | - |
| At4g39361<br>At4g39363 | snoRNA cluster | -199 | -300,-323 | -140 |

| | | | | |
|---|---|---|---|---|
| At4g39364<br>At4g39366 | | | | |
| At5g10572 | snoRNA | -353 | -444,-469,-471 | EST |
| At5g13225 | snoRNA | -236 | -267,-299,-305 | EST |
| At5g44286 | snoRNA | -326 | -454,-490 | - |
| At5g51174 | snoRNA | -311,-384 | -412 | EST |
| At5g52471 | snoRNA, nested within fibrillarin gene | -130 from ATG | -285,-300 from ATG | -103 from ATG |
| At5g58595 | snoRNA | -598,-715 | -569,-741,-766,-785 | -671 |
| At5g66562<br>At5g66564<br>At5g66567 | snoRNA cluster | -138,-281 | -495,-470 | - |
