# Supplementary material for: Distribution of short interstitial telomere motifs in two plant genomes: putative origin and function
Source: BMC Plant Biol. 2010 Dec 20;10:283. doi: 10.1186/1471-2229-10-283 (PMC3022908; doi:10.1186/1471-2229-10-283)
Supplement: Additional File 4 — This file contains a table showing in O. sativa the location of telo boxes, site II motifs, TEF1 boxes and transcription start sites of snoRNA precursors relative to the 5' end of the first mature snoRNA in independent clusters, the 5' end of the mature orphan snoRNA or relative to the translation initiation codon when snoRNA genes are nested within a protein coding gene. [file 1471-2229-10-283-S4.PDF]

Additional File 4

O. sativa snoRNA genes. The location of *telo* boxes, site II motifs, TEF boxes and TSS is indicated relative to the 5' end of the mature snoRNA (first snoRNA when found in an independent cluster) or relative to the translation initiation codon of genes harbouring intron-encoded snoRNAs.

Locus	**product**	***telo* box**	*Site II or TEF*	**5' mRNA**
Cluster 1	snoRNA cluster nested within ribosomal protein S10	-31	-251,-284	-119
Cluster 2	snoRNA cluster	-172,-247,-257	-14,-42,-313,-337,-351	-194
Cluster 3	snoRNA cluster	-236	-298,-314,-336	-
Cluster 4	snoRNA cluster nested within 60S ribosomal protein L30	-98	-171,-177,-210	-86
Cluster 5	snoRNA cluster nested within ribosomal protein L41	-152	-235,-271,-835,-972	-94
Cluster 6	snoRNA cluster nested	-802	-832,-845,-892,-907,-957	- 96
Cluster 7	snoRNA cluster	-254	-265,-281	
Cluster 8	snoRNA cluster	-391,-898		-445
Cluster 9	snoRNA cluster nested within hypothetical protein	-580	-52,-82,-401,-412, -359 (TEF)	-
Cluster 10	snoRNA cluster	-254	-325,-337	-201
Cluster 12	snoRNA cluster		-340,-350,-391	-
Cluster 13	snoRNA cluster nested within ribosomal protein L41	-152	-233,-271,-835,-972	-
Cluster 14	snoRNA cluster nested within 60S ribosomal protein L23a	-138	-148,-191,-200,-220	-88
Cluster 16	snoRNA cluster	-208	-239,-259,-286,-311	-151
Cluster 18	snoRNA cluster	-346	-376,-389436,-451,-501	-328
Cluster 15-17-19	snoRNA cluster nested within 60S ribosomal protein L13a	-243,-297	-353,-365	-276
Cluster 20	snoRNA cluster	-	-1346,-1399,-14261447,-1453,-1524, -328 (TEF)	-236
Cluster 21	snoRNA cluster nested within snRNP-G	-	-169,-262	-127
Cluster 22	snoRNA cluster nested within snRNP-G	-142	-158,-173,-210,-225,-243	-73
Cluster 23	snoRNA cluster	-185	-223,-229,-248,-275	-137
Cluster 25	snoRNA cluster	-237	-263,-274	-
Cluster 27	snoRNA cluster nested	-173	-203,-209,-678	-65
Cluster 28	snoRNA cluster	-246		-
Cluster 30	snoRNA cluster	-192	-222,-234,-267	-138
Cluster 31	snoRNA cluster nested within 40S ribosomal protein S9	-1065	-1085,-1116,-1129	-1019
Cluster 32	snoRNA cluster nested within 40S ribosomal protein S9	-866	-895,-930,-942	-94
Cluster 33	snoRNA cluster	-	-	-
Cluster 34	snoRNA cluster	-	-	-
Cluster 35	snoRNA cluster	-	-	-
Cluster 36	snoRNA cluster	-	-	-
Cluster 37	snoRNA cluster	-	-	-
Cluster 38	snoRNA cluster	-	-232,-257,-279	-
Cluster 39	snoRNA cluster nested within eukaryotic TIF 2 beta	-193	-300,-355,-388	-283
Cluster 40	snoRNA cluster	-	-	-
Cluster 42	snoRNA cluster	-242	341,-362,-371,-383,-625	-192
Cluster 43	snoRNA cluster nested within 60S ribosomal protein L34	-	-	-57
Cluster 44	snoRNA cluster	-	553,-564,-578,-878	-500
Cluster 45	snoRNA cluster nested within Heat shock protein 70	-40	-228,-279,-299	-77

Cluster 47	snoRNA cluster nested within ribosomal protein L37e	-155	-195,-205,-233,-271,-291	-315
Cluster 48	snoRNA cluster nested within 60S ribosomal protein L37A	-168	-208,-218,-245	-314
Cluster 49	snoRNA cluster	-	-	-
Cluster 50	snoRNA cluster	-274	320,-326,-339,-354	-
Cluster 51	snoRNA cluster Non-protein coding transcript, unclassifiable	-437	-494,-520,-538,-562,-570,-577	-429
Cluster 52	snoRNA cluster nested within Ubiquitin fused to ribosomal protein L40	-127	-208,-231	-129
Cluster 53	snoRNA cluster	-265	308	-
Cluster 54	snoRNA cluster	-	454	-
Cluster 55	snoRNA cluster	-553	596,-601,-632	-497
Cluster 56	snoRNA cluster	-192,-252	292,-298,-317	-189
Cluster 57	snoRNA cluster nested within ribosomal protein L28e	-880	-906,-916,-946	-830
Cluster 58	snoRNA cluster nested within 60S ribosomal protein L18	-220	-122,-236,-245,-279,-289,-311,-415	-129
Cluster 59	snoRNA cluster nested within hypothetical protein	-822	-283	-81
Cluster 60	snoRNA cluster nested within ribosomal protein L28e	-615	-674,-692,-734,-744,-750	-569
Cluster 61	snoRNA cluster	-	-	-
Cluster 62	snoRNA cluster	-619	686,-732,-754	-597
Cluster 63	snoRNA cluster		661,-670,-690	-572
Cluster 64	snoRNA cluster	-218	236,-245,-818,-1032	-172
Cluster 65	snoRNA cluster	-	-275,-284,-308,-326,-612	-
Cluster 66	snoRNA cluster	-	-275,-1064	-981
Cluster 67	snoRNA cluster nested within TGF-beta receptor-interacting protein 1	-80	-166,-177	-124
Cluster 68	snoRNA cluster	-	-	-
Cluster 69	snoRNA cluster	-821	-833,-868	-
Cluster 70	snoRNA cluster nested within TGF-beta receptor-interacting protein 1	-107	-189,-415	-143
Cluster 71				
Cluster 72				
Cluster 73	snoRNA cluster	-	-	
Cluster 74	snoRNA cluster	-	-	
snoR68Y	snoRNA	-229	-403,-415,-439	-267
snoR19	snoRNA nested within hypothetical protein	-444	-453,-490	-379
snoR21	snoRNA	-	-337,-345,-372,-383	-
snoR21	snoRNA	-252	-299,-354,-373,-386,-395	-
snoR60 snoR60	snoRNA cluster nested within fibrillarin fragment	-1297,-1396,-1415,-1652	-1826,-1838,-1862	-1026
snoR60 snoR60	snoRNA cluster nested within fibrillarin	-62,-80,+28	-	-
snoR66a	snoRNA	-	-	
snoR66b	snoRNA	-	-	
snoR66d	snoRNA nested within 60S ribosomal protein L44	-31,-137	-166,-181,-187	
snoR66c	snoRNA	-945	-1052,-1064	
snoR66e	snoRNA	-609	-764,-775,-782	
snoR66f	snoRNA	-945	-1052,-1064	
snoR66g	snoRNA	-945	-1052,-1064	
snoR66h	snoRNA	-945	-1052,-1064	
snoR66i	snoRNA	-622	-732,-738,-776,-797,-803	
snoR66j	snoRNA	-619	-729,-735,-797,-803	
snoR66k	snoRNA	-619	-729,-735,-794,-800	
snoR120	snoRNA	-594	-693,-714,-723,-737,-977	-520
snoR124	snoRNA	-1060	-	-
snoR125	snoRNA nested within Ribosomal protein L9 family	-465	-	-
snoR127	snoRNA	-	-	-

snoR128	snoRNA	-	-	-
snoR144	snoRNA	-	-	-
snoR148	snoRNA	-	-484	-
snoR154	snoRNA	-	-374	
snoR154	snoRNA	-	-41	
snoR154	snoRNA	-	-732	
snoR154	snoRNA	-	-	-
snoR156	snoRNA	-	-	-
snoR158	snoRNA	-	-	-
snoR161	snoRNA	-	-161,-175,-191,-206	-
snoR161	snoRNA	-	-143,-161	-
snoR165	snoRNA	-	-	-
snoR169	snoRNA	-	-	-
snoR170	snoRNA	-	-	-
snoR171a	snoRNA	-	-	
snoR171b	snoRNA	-	-	
snoR172	snoRNA	-267,-786	-	
snoR176	snoRNA	-120	-136,-145,-179,-211,-315,-703	
snoR178	snoRNA nested within conserved hypothetical protein	-262	-48,-131,-141,-158,-168,-193,-684	
snoR165	snoRNA			
snoR142b snoR142c	cluster of snoRNA	-821	-833,-868,-1186,-1220	
U59	snoRNA	-	-447,-739	-
U59	snoRNA	-	-	-
U34	snoRNA nested	-214		
