# Supplementary material for: Distribution of short interstitial telomere motifs in two plant genomes: putative origin and function
Source: BMC Plant Biol. 2010 Dec 20;10:283. doi: 10.1186/1471-2229-10-283 (PMC3022908; doi:10.1186/1471-2229-10-283)
Supplement: Additional File 5 — This file contains a table giving the chromosomic location and positions of snoRNAs. [file 1471-2229-10-283-S5.PDF]

Additional File 5

Nature and location of O. sativa snoRNA transcript units analysed in this work.

Red: snoRNA not found in clusters
Green: snoRNA that could form new clusters

**Cluster Name**	**Type**	**Chr**	**snoRNA**	**Positions**
Cluster 2	Intergenic	1	snoR15a-snoR18a-snoR28h	9656206-9654537
Cluster 4	Intronic	1	snoR28e-snoR28f-snoR28g	9661165-9657150
Cluster 9	Intronic (?)	1	snoR122a-snoR122b-snoR72Yb-snoR123-snoR72Ya	12907495-12904473
Cluster 14	Intronic	1	U15d-snoR7c	13878260-13880999
Cluster 23	Intergenic	1	snoR41Y1a-snoR36a	41004197-41002939
Cluster 49	Intergenic	1	snoR162a-snoR162b	145149-143909
Cluster 6	located at +440 of a coding sequence	2	snoR29a-snoR30a-snoR31a-snoR10a-snoR77Ya-U49a-snoR2a-U14a-U14b-U14cψ	26280929-26283528
Cluster 8	Intergenic	2	snoR121a-snoR121b-snoR121c-snoR121d-snoR121e-snoR121fψ-snoR121g-snoR121h	785757-782338
Cluster 20	Intergenic	2	snoR22a-snoR23-snoR22b	5510333-5509231
Cluster 47	Intronic	2	snoR159a-U18c-snoR13a-snoR58Ya-snoR13b	632726-635778
Cluster 50	Intergenic	2	U61-snoR14	10333158-10334470
Cluster 55	Intergenic	2	snoR160a-snoR160b-snoR160c-snoR160d-snoR160eψ-snoR160f	30290082-30292290
Cluster 57	Intronic	2	snoR24f-snoR24g	35276850-35274662
Cluster 65	Intergenic	2	snoR1a-snoR1b-snoR1c	1799101-1797450
snoR19d		2	snoR19d	24582822-24583905
Cluster 71	5' UTR	2	**snoR60-snoR60**	35289092-35293260
Cluster 3	Intergenic	3	snoR28a-snoR28b-snoR28c-snoR28d	1673159-1671448
Cluster 5	Intronic	3	U15a-U15b-snoR7a-U18a	32187326-32185219
Cluster 15-17.2	Intergenic	3	- snoR2c-U14d-U14e-U14fψ-snoR29b-snoR30b-snoR31b-snoR10b-snoR77Yb-U49b-snoR2b-U14gψ-U14hψ-snoR29c<br>- snoR29eψ-snoR30c-snoR31c-snoR10c-snoR77Yc-U49c-snoR2e	30975901-30971609
Cluster 17.1	Intronic	3	snoR12a-U24a-snoR29eψ-snoR30c-snoR31c-snoR10c-snoR77Yc-U49c-snoR2e	30978154-30975910
Cluster 19	Intronic	3	snoR12b-U24b	30989002-30991992
Cluster 22		3	U40c-U40d	16746103-16749042
Cluster 27	Intergenic, just after the protein	3	snoR37a-snoR37b-snoR64a-snoR38Ya-snoR20cψ-U50a	16592133-16595204
Cluster 32	Intronic	3	U35c-snoR132b	14838435-14840975

Cluster 38 not a cluster	Intergenic	3	snoR146a-snoR146b-snoR146c-snoR146d	24266278-24265127 only the first snoRNA was found
Cluster 39	Intronic	3	U34a-U34b-U34c-U34d-U34e	12284874-12290564
Cluster 52	Intronic	3	snoR142a-snoR142b	7073110-7069653
Cluster 64	Intergenic	3	U43a-snoR130-snoR16b-snoR16a	5128587-5126801
Cluster 66	Intergenic	3	U43b-snoR16c	12689617-12687986
Cluster 7	Intergenic	4	snoR13e-U18d-snoR58Yc	29123758-29125161
Cluster 16	Intergenic	4	snoR29d-snoR2d-U14i-U14j-U14k	27211291-27213404
Cluster 59	Intronic	4	snoR19a-snoR19eψ-snoR19b-snoR19c	25357947-25362185
Cluster 25	Intergenic	5	snoR41Y1c-snoR36c	17710271-17708951
Cluster 42	Intergenic	5	snoR32-U27a-snoR120-U80a	26070381-26068739
Cluster 58	Intronic	5	snoR53Y-U29a-U29b	3169069-3172525
Cluster 67	Intronic	5	U54a-U54b-U54c-U54d	9347559-9354090
Cluster 60	Intronic	5	snoR24a-snoR24b-snoR24c-snoR24d-snoR24e	26698650-26695222
Cluster 72	Intronic	5	**snoR60-snoR60**	4499684-4503743
Cluster 1	Intronic	6	U36Ia-U36Ib-U36Ic-U36Id	1821923-1817466
Cluster 12	Intergenic	6	snoR15b-snoR18b	19711492-19712785
Cluster 30	Intergenic	6	snoR37c-snoR37d-snoR64b-snoR38Yb-snoR20b	28062020-28064003
Cluster 56	Intergenic	6	snoR167-snoR47	28822909-28824208
Cluster 54	Intergenic	6	snoR158a-snoR147b-snoR158b-snoR158c	6794041-6796831]
Cluster 63	Intergenic	6	U36IIc-U38e-snoR126c-U38f	27880422-27882984
Cluster 13	Intronic (?)	7	U15c-snoR7b-U18b (U18b not found)	2126670-2125433
Cluster 21	Intronic	7	U40a-U40b	24986374-24983325
Cluster 31	Intronic	7	U35a-U35b-snoR132a	25985714-25982861
Cluster 51	Intronic	7	snoR12c-U24c-snoR12d-U24d	3703866-3706565
Cluster 61	Intergenic	7	U36IIb-U38b-snoR126b	2063226-2061797
Cluster 62	Intergenic	7	U36IIa-U38a-snoR126a	2064469,2063054
Cluster 48	Intronic	8	snoR159b-snoR13c-snoR58Yb-snoR13d	1608442-1614876
Cluster 73	Intergenic	7	**snoR166-snoR166**	21093120-21091763
Cluster 70	Intronic	8	**U54-U54-U54**	12907272-12899724
Cluster 43	Intronic	9	U27b-U80b	3285787-3289545
Cluster 53	Intergenic	9	snoR44-snoR17-snoR147a	14572201-14570690
Cluster 69	Intergenic	9	**snoR142-snoR142**	22391053-22392534
Cluster 10	Intergenic	10	snoR39BYa-snoR39BYb-snoR39BYc	11062911-11064425
Cluster 33	Intergenic	10	snoR133a-snoR133b-snoR134a	12260305-12262217
Cluster 34.1 et Cluster 34.2	Intergenic	10	- snoR134b-snoR137a-snoR136b-snoR138<br>- snoR137b-snoR139	12281772-12285229
Cluster 35-36-37	intergenic	10	- snoR141a-snoR129a-snoR137e-snoR137c-snoR143a-snoR137f-snoR145d	12276000-12284000

			- snoR140b-snoR141b-snoR129b<br>- snoR145a-snoR143c	
Cluster 41	Intergenic	10	snoR149b-snoR150b-snoR151a-snoR152-snoR149c-snoR150c-snoR151b-snoR153	12347619,12352002
Cluster 74	Intergenic	10	**snoR166-snoR166**	8883733-8882466
Cluster 45	Intronic	11	U33c-U51d-snoR5c-U33d-U51e-snoR5d	28172149-28177441
U38A	Intergenic	10	U38A	4412371-4413464
Cluster 68	Intergenic/Intergenic	11	U54e-U54f-U54g-U54h-U54i	22544825-22547510
Cluster 44.1 and Cluster 44.2	Intergenic	12	- snoR157a-U33a-U51a-snoR5a-U51b-snoR5g<br>- snoR157b-U33b-U51c-snoR5b	23475314-23478749
Cluster 28	Intergenic	12	U59b-snoR41YIIa-U59c	22550439-22552051
Cluster 18	Not found		U14l-U14m-U14nψ	Not found. Only three locations on chr2 (C6 cluster), chr3 (C15-C17 cluster) and chr4 (C16 cluster)
Cluster 11	Not found		snoR39BYd-snoR39BYe	Not found with given accession numbers: exactly similar to snoR39BYb and snoR39BYc
Cluster 24	Not found		snoR41Y1b-snoR36b	Similar to Cluster 23 or Cluster 25
Cluster 26	Repeats (more than 100)		snoR131a-snoR131b-snoR131c	More than 100 repeats found with given accession numbers
Cluster 40	Not found		snoR149a-snoR150a	
Cluster 46	Not found		U51f-snoR5f-U33e-U51g-snoR5e	
Cluster 29	Not found		U59d-snoR41YIIb-U59e	

chromosomic location snoRNA clusters

snoRNA name	Intergenic/Intronic	Chromosome	Positions
snoR21	Intergenic	1	15698780-15699839
snoR170	Intergenic	1	34383023-34381907
snoR173d	Intergenic	1	8970360-8971491
snoR19	3' end of a hypothetical protein	2	24582828-24583885
snoR21	Intergenic	2	21042684-21043771
snoR165	Intergenic	2	28754304-28753234
snoR173c	Intergenic	2	26936-28066
snoR178	Intronic (?)	2	1883475-1879323
U38A	Intergenic	3	6372424-6371331
snoR39BY	Intergenic	3	17380319-17378752
snoR66c*	Intergenic	3	11055531-11056459
snoR169	Intergenic	3	31706859-31705693
snoR158	Intergenic	4	21230142-21228039
snoR148	Intergenic	5	20394247,20395316

snoR127	Intergenic	5	25145372-25146462
snoR120	Intergenic	5	26070029-26068964
snoR66a*	Intergenic	5	2952275-2951198
snoR66b*	Intergenic	5	2950627-2949552
snoR176a	Intronic	5	3168916-3172525
snoR177*a	Intergenic	5	3170555-3169449
snoR177*b	Intergenic	5	13562998-13561895
U59	Intergenic	5	14473380-14472310
snoR144	Intergenic	6	27586969-27588056
snoR66d*	Intronic	7	20255534-20258289
snoR66e*	Intergenic	7	20207285-20208352
snoR66f*	Intergenic	7	20220018-20221085
snoR66g*	Intergenic	7	20238235-20239302
snoR66h*	Intergenic	7	20256453-20257520
snoR66i*	Intergenic	7	20213846-20214909
snoR66j*	Intergenic	7	20232063-20233126
snoR66k*	Intergenic	7	20250282,20251345
U34	Intronic	7	28810358-28806850
snoR163	Intergenic	8	19781402-19780271
snoR163	Intergenic	8	19769568-19768439
snoR163	Intergenic	8	19757151-19756034
snoR163	Intergenic	8	19760337-19759208
snoR171a	Intergenic	8	4031243-4030050
snoR172	Intronic	8	5581353-5578088
snoR173a	Intergenic	8	10665960-10667090
snoR125	Intronic (?)	10	11064613-11069583
snoR124	Intergenic	10	11061069-11059939
U38A	Intergenic	10	4412371-4413430
snoR68Y	Intergenic	10	18580900-18579841
snoR154	Intergenic	10	12359538-12360661
snoR154	Intergenic	10	12365032-12366110
snoR154	Intergenic	10	12366047-12367161
snoR154	Intergenic	10	12365780-12366894
snoR154	Intergenic	10	12359977-12361048
snoR156	Intronic	10	12358440-12354309
snoR173b	Intergenic	10	15349301-15350431
snoR135	Intergenic	10	12284913-12285995
snoR161	Intergenic	11	5818274-5817165
snoR128	Intergenic	11	15020447-15021556
snoR161	Intergenic	12	4697376-4696265
U35A	Not found		
snoR52Y	Not found		
snoR140b	Not found		
snoR143c	Not found		
snoR145	Not found		

snoR155	Not found		
snoR164	Not found		
snoR168	Not found		
